# Dataset and analysis of automated and manual methods to differentiate wide QRS complex tachycardias

**DOI:** 10.1016/j.dib.2024.111198

**Published:** 2024-12-03

**Authors:** Sarah LoCoco, Anthony H. Kashou, Abhishek J. Deshmukh, Samuel J. Asirvatham, Christopher V. DeSimone, Krasimira M. Mikhova, Sandeep S. Sodhi, Phillip S. Cuculich, Rugheed Ghadban, Daniel H. Cooper, Thomas M. Maddox, Peter A. Noseworthy, Adam M. May

**Affiliations:** a Department of Medicine, Division of Cardiovascular Diseases, Washington University School of Medicine in St. Louis, St. Louis, MO, United States; b Department of Cardiovascular Medicine, Mayo Clinic, Rochester, MN, United States

**Keywords:** Wide QRS complex tachycardia, Wide complex tachycardia, Ventricular tachycardia, Supraventricular wide complex tachycardia, Automated algorithms

## Abstract

The differentiation of wide complex tachycardias (WCTs) into ventricular tachycardia (VT) and supraventricular wide tachycardia (SWCT) via 12-lead ECG (electrocardiogram) interpretation is a crucial yet demanding clinical task. Decades of research have been dedicated to simplifying and improving this differentiation via manual algorithms. Despite such research, the effectiveness of such algorithms still remains limited, primarily due to reliance on user expertise. To combat this limitation, automated algorithms have been created that show promise as alternatives to manual ECG interpretation. However, direct comparison of the methods’ diagnostic performances has not been undertaken. A recent publication (LoCoco et al., 2024) compared the diagnostic performance between traditional manual ECG interpretation approaches (i.e. Brugada, Vereckei aVR, and VT Score) to novel automated wide QRS complex tachycardia differentiation algorithms (i.e. WCT Formula I, WCT Formula II, VT Prediction Model, Solo Model, and Paired Model). Two electrophysiologists independently applied the 3 manual WCT differentiation approaches to 213 ECGs. Simultaneously, computerized data from the same paired WCT with baseline ECGs were processed by the 5 automated WCT differentiation algorithms. Following these analyses, the diagnostic performance of automated algorithms was compared with manual ECG interpretation approaches. In this article, a summary of data components relating to diagnostic performance of the methods tested is presented.

Specifications Table

**Table:** 

Subject	Cardiology
Specific subject area	Electrophysiology, electrocardiography
Type of data	Tables and figures of analyzed data
Data collection	Review of health records, manual electrocardiogram interpretations by expert electrophysiologists via manual interpretation algorithms, and automated measurements provided by computerized electrocardiogram interpretation software
Data source location	Barnes-Jewish Hospital in St. Louis via Washington University School of Medicine in St. Louis, Missouri
Data accessibility	Featured data within this article DOI: https://doi.org/10.7910/DVN/VMYTJR
Related research article	LoCoco S, Kashou AH, Deshmukh AJ, Asirvatham SJ, DeSimone CV, Mikhova KM, Sodhi SS, Cuculich PS, Ghadban R, Cooper DH, Maddox TM, Noseworthy PA, May AM. Direct Comparison of Methods to Differentiate Wide Complex Tachycardias: Novel Automated Algorithms Versus Manual ECG Interpretation Approaches. Circ Arrhythm Electrophysiol. 2024 Aug;17(8):e012663. doi: 10.1161/CIRCEP.123.012663. Epub 2024 Jul 25. PMID: 39051111.

## 1 Value of the Data

• Data would be valuable to researchers interested in comparing the diagnostic performance of novel automated electrocardiogram tools to traditional manual algorithms in differentiating wide QRS complex tachycardias (WCTs) as ventricular tachycardia (VT) or supraventricular wide complex tachycardia (SWCT).
• Data would be valuable to researchers interested in the performance of traditional manual methods and novel automated algorithms for WCT differentiation.
• Enclosed data describes the logistic regression model structure of the five automated WCT differentiation methods.
• Enclosed data summarizes the diagnostic performance amongst manual WCT differentiation methods.
• Enclosed data summarizes the diagnostic performance amongst novel automated WCT differentiation methods.
• Enclosed data details the specific diagnostic performance metrics of manual algorithm components as applied by two expert electrophysiologists.

## 2 Background

In this article, a summary of data components relating to diagnostic performance of the methods tested is presented for a recent publication (3) comparing the diagnostic performance of traditional manual to novel automated ECG interpretation approaches. This data expands on the data, analytic methods, and study materials utilized in order to allow other researchers for opportunity of reproducing the results by replicating the procedure.

## 3 Data Description

1. Table 1 outlines the parameters utilized by automated algorithms, listing and describing each individual parameter, highlighting the source ECG(s) for each feature, and specifying the algorithms that employ them. Additionally, the table summarizes the electrophysiological basis for parameter development and application. For example, the rationale for calculating the absolute change in QRS duration stems from the understanding that VT typically originates and depolarizes outside the heart's native His-Purkinje pathways, resulting in larger QRS duration changes compared to the baseline rhythm, unlike SWCT (1,2). The table also classifies parameters as either 'measured' or 'engineered,' indicating whether the parameter is a direct computerized ECG measurement or a mathematically derived value based on computerized ECG measurements.

**Table 1:** Automated algorithm parameters.

Parameter Name	Measured or Engineered	Parameter Description	Electrophysiologic Rationale	Source ECG(s)	Algorithms
WCT QRS duration (ms)	Measured	Direct measurement of QRS duration of the WCT ECG.	The less efficient ventricular depolarization in VT typically leads to longer QRS durations compared to SWCT.	WCT ECG only	WCT Formula VT Prediction Model WCT Formula II Solo Model Paired Model
Baseline QRS duration (ms)	Measured	Direct measurement of QRS duration of the baseline ECG.	Patients with conduction delays, often due to myocardial disease, typically have longer baseline QRS durations. Baseline QRS duration is generally more pronounced in patients with VT compared to those with SWCT.	Baseline ECG only	VT Prediction Model WCT Formula II
Change in QRS duration (ms)	Engineered	Absolute arithmetical difference in QRS duration (ms) measurements between paired WCT and baseline ECGs.	VT typically originates and depolarizes outside the heart's native His-Purkinje pathways, causing greater changes in QRS duration compared to the baseline rhythm. In contrast, SWCT often exhibits ventricular depolarization more similar to that of the baseline rhythm, resulting in only minor changes in QRS duration.	Paired WCT and Baseline ECGs	VT Prediction Model
Change in QRS axis (°)	Engineered	Quantification of degree (°) of change in the mean electrical axis of ventricular depolarization.	VT onset can lead to varied depolarization in the ventricular myocardium, causing significant shifts in the electrical axis compared to the baseline rhythm. In contrast, SWCTs typically utilize similar pathways as the baseline rhythm, resulting in only minor changes in the QRS axis.	Paired WCT and Baseline ECGs	VT Prediction Model
Change in T wave axis (°)	Engineered	Quantification of degree (°) of change in the mean electrical axis of ventricular repolarization.	VT onset typically causes substantial changes in ventricular repolarization, while SWCTs generally result in smaller changes.	Paired WCT and Baseline ECGs	VT Prediction Model Paired Model
Frontal PAC (%)	Engineered	Quantification of the degree of QRS amplitude change (%) between paired WCT and baseline ECGs in select leads of the frontal plane (aVR, aVL, and aVF).	Significant depolarization shifts during the onset or offset of WCT often suggest VT, due to its diverse origins and broad mechanisms of ventricular myocardium depolarization. In contrast, minor changes typically indicate SWCT, which primarily follows the same conduction pathways as the baseline heart rhythm.	Paired WCT and Baseline ECGs	WCT Formula Paired Model
Horizontal PAC (%)	Engineered	Quantification of the degree of QRS amplitude change (%) between paired WCT and baseline ECGs in select leads of the horizontal plane (V1, V4, and V6).	Significant depolarization shifts during the onset or offset of WCT often suggest VT, due to its diverse origins and broad mechanisms of ventricular myocardium depolarization. In contrast, minor changes typically indicate SWCT, which primarily follows the same conduction pathways as the baseline heart rhythm.	Paired WCT and Baseline ECGs	WCT Formula Paired Model
Frontal PTVAC (%)	Engineered	Quantification of the degree of QRS time-voltage area change (%) between paired WCT and baseline ECGs in select leads of the frontal plane (aVR, aVL, and aVF).	Large shifts in depolarization at the onset or offset of WCT typically indicate VT, due to its diverse origins and broad mechanisms of ventricular myocardium depolarization. In contrast, minor changes suggest SWCT, which predominantly utilizes the same conduction pathways as the baseline heart rhythm.	Paired WCT and Baseline ECGs	WCT Formula II Paired Model
Horizontal PTVAC (%)	Engineered	Quantification of the degree of QRS time-voltage area change (%) between paired WCT and baseline ECGs in select leads of the horizontal plane (V1, V4, and V6).	Large shifts in depolarization at the onset or offset of WCT typically indicate VT, due to its diverse origins and broad mechanisms of ventricular myocardium depolarization. In contrast, minor changes suggest SWCT, which predominantly utilizes the same conduction pathways as the baseline heart rhythm.	Paired WCT and Baseline ECGs	WCT Formula II Paired Model
PMonoTVA (%)	Engineered	Calculated percentage (%) of QRS TVA contained by monophasic (as opposed to multiphasic) QRS complexes on the WCT ECG.	VT often depolarizes uniformly from its origin, bypassing the native His-Purkinje system and producing monophasic QRS complexes on the ECG. In contrast, most SWCTs originate from specialized conduction tissue, leading to multiphasic QRS complexes.	WCT ECG only	Solo Model Paired Model

Summary of the parameters utilized by automated algorithms, listing and describing each individual parameter, highlighting the source ECG(s) for each feature, and specifying the algorithms that employ them. *ECG, electrocardiogram; PMonoTVA, percent monophasic time-voltage area; PAC, percent amplitude change; PTVAC, percent time-voltage area change; SWCT, supraventricular wide complex tachycardia; TVA, time-voltage area; VT, ventricular tachycardia; WCT, wide complex tachycardia*
2. As noted in the original work (3), two electrophysiologists applied three manual WCT differentiation approaches (Brugada algorithm (4), Vereckei aVR algorithm (5), and VT score (6) to 213 WCT ECGs (for a total of 426 separate interpretations per algorithm). Table 2 summarizes the comparison of diagnostic performance between manual methods, classifying the performance as equivalent, inferior, or superior in terms of accuracy, sensitivity, specificity, and F1 score. Significant between-group differences were classified as either inferior or superior via the utilization of non-overlapping confidence intervals.

**Table 2:** Manual method comparisons.

Manual Method Comparison	Accuracy	Sensitivity	Specificity	F1 score
Brugada vs. Vereckei aVR	Equivalent	Equivalent	*Superior*	Equivalent
Brugada vs. VT score	Equivalent	Equivalent	*Inferior*	Equivalent
Vereckei aVR vs. VT score	*Inferior*	Equivalent	*Inferior*	*Inferior*

Summary of diagnostic performance comparisons between manual methods. Non-overlapping confidence intervals were used to define significant between-group differences (i.e., superior or inferior). *VT, ventricular tachycardia*
3. Table 3 summarizes the diagnostic performance comparisons between the five automated algorithms (WCT Formula (7), WCT Formula II (8), VT Prediction Model (1), Solo Model (9), and Paired Model (9)). Comparisons were categorized as equivalent, inferior, or superior based on accuracy, sensitivity, specificity, and F1 score. Significant differences between groups were classified as either inferior or superior using non-overlapping confidence intervals.

**Table 3:** Automated algorithm comparisons.

Automated Algorithm Comparison	Accuracy	Sensitivity	Specificity	F1 score
WCT Formula vs. VT prediction Model	Equivalent	Equivalent	Equivalent	Equivalent
WCT Formula vs. WCT Formula II	Equivalent	Equivalent	Equivalent	Equivalent
WCT Formula vs. Solo Model	Equivalent	Equivalent	Equivalent	Equivalent
WCT Formula vs. Paired Model	Equivalent	Equivalent	Inferior	Equivalent
VT prediction Model vs. WCT Formula II	Inferior	Inferior	Equivalent	Inferior
VT prediction Model vs. Solo Model	Equivalent	Equivalent	Equivalent	Equivalent
VT prediction Model vs. Paired Model	Inferior	Equivalent	Inferior	Inferior
WCT Formula II vs. Solo Model	Equivalent	Superior	Equivalent	Equivalent
WCT Formula II vs. Paired Model	Equivalent	Equivalent	Inferior	Equivalent
Solo Model vs. Paired Model	Equivalent	Equivalent	Equivalent	Equivalent

Summary of diagnostic performance comparisons between automated algorithms. Non-overlapping confidence intervals were used to define significant between-group differences (i.e., superior or inferior). *WCT, wide complex tachycardia; VT, ventricular tachycardia*
4. Table 4 demonstrates the frequency each step of the VT score was utilized by the two electrophysiologists.

**Table 4:** Frequency of use for VT Score criteria.

VT Score Criteria		Frequency
**#1**	*Initial dominant R in lead V1!?*	26.8% (114 of 426)
**#2**	*Initial r > 40 ms in lead V1 or V2?*	10.8% (46 of 426)
**#3**	*Notched S in lead V1?*	10.6% (45 of 426)
**#4**	*Presence of an initial R wave?*	28.6% (122 of 426)
**#5**	*Lead II R wave to peak time (RWPT) ≥ 50 ms?*	30.5% (130 of 426)
**#6**	*Absence of an RS complex in leads V1-V6?*	15.2% (65 of 426)
**#7**	*AV dissociation?*	11.5% (49 of 426)

Summary of frequency each criterion of VT Score was utilized by the two electrophysiologists. *AV, atrioventricular; RWPT, R wave to peak time.*
5. Table 5 highlights the diagnostic performance in terms of positive predictive value (PPV) and negative predictive valve (NPV) for total point scores attained using the VT score.

**Table 5:** Predictive value of VT Score.

VT Score	Frequency	PPV	NPV
**0 points**	166 of 426 (38.9%)	NA	90.9%
**≥ 1 points**	260 of 426 (61%)	57.1%	48.9%
**≥ 2 points**	190 of 426 (44.6%)	85%	55.5%
**≥ 3 points**	110 of 426 (25.8%)	91.8%	54.5%
**≥ 4 points**	49 of 426 (11.5%)	91.6%	51.5%
**≥ 5 points**	13 of 426 (0.03%)	52.1%	100%
**≥ 6 points**	7 of 426 (0.02%)	85.7%	48.5%
**≥ 7 points**	0 of 426 (0%)	NA	NA

**Fig. 1:**
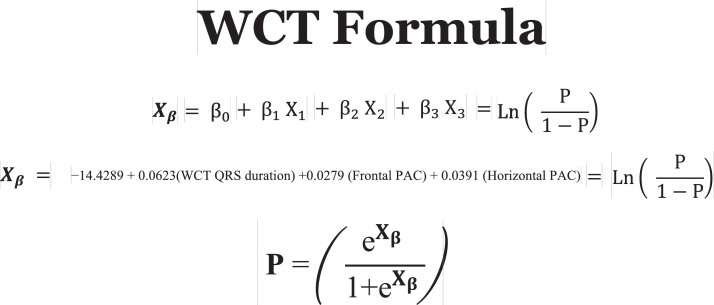
Logistic regression structure of the WCT formula.

**Fig. 2:**
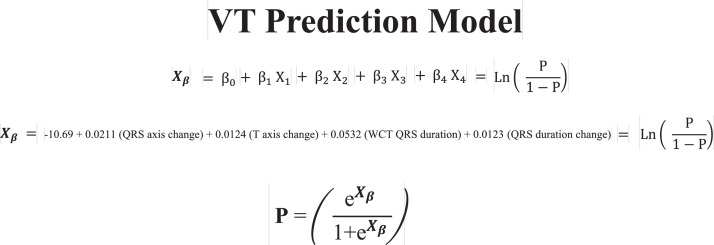
Logistic regression structure of the VT prediction model.

**Fig. 3:**
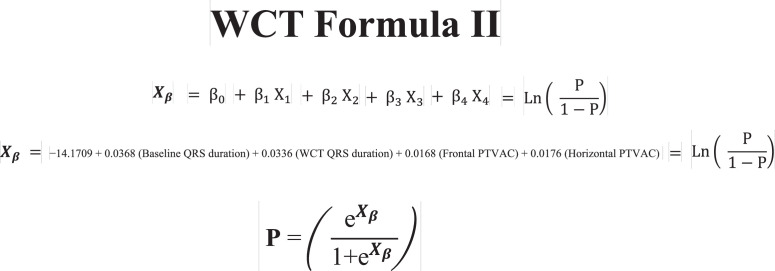
Logistic regression structure of theWCT Formula II.

**Fig. 4:**
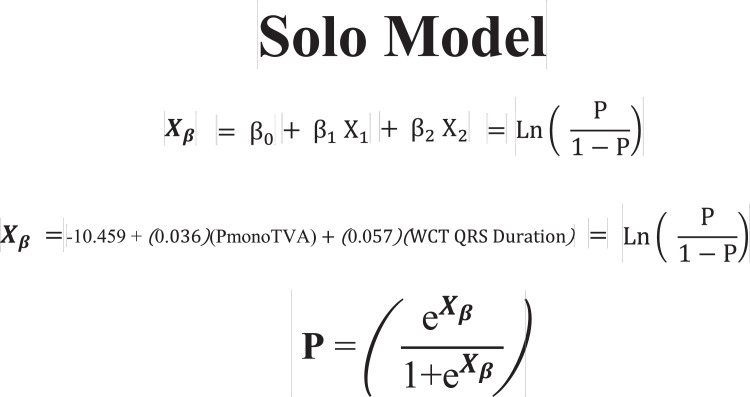
Logistic regression structure of the Solo model.

**Fig. 5:**
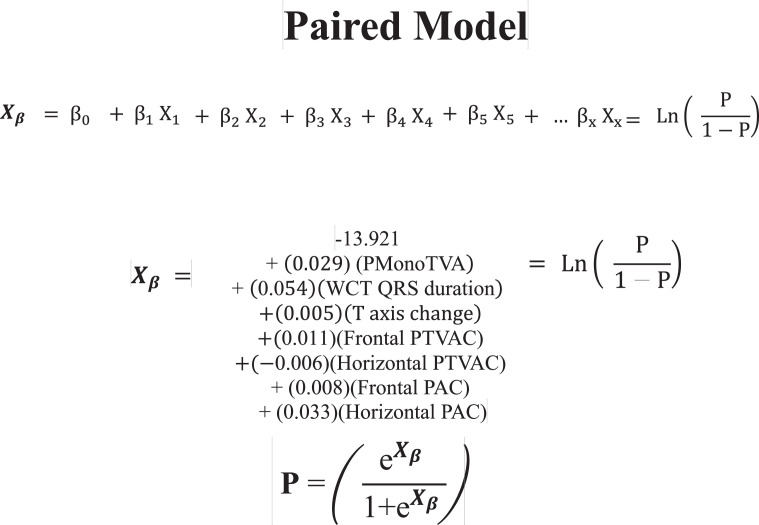
Logistic regression structure of the paired model.

Summary of the diagnostic performance various point totals obtained via VT Score application. *PPV= positive predictive value; NA, non-applicable; NPV= negative predictive value*
6. Fig. 1, Fig. 2, Fig. 3, Fig. 4, Fig. 5 illustrate the logistic regression models used in the WCT Formula, VT Prediction Model, WCT Formula II, and Solo Model, respectively. Each logistic regression model assigns a beta coefficient (βx) to parameters comprising the model based on their influence on the binary outcome (i.e., VT or SWCT). Parameters, as detailed in Table 1, are key determinates influencing the predictive performance of each model.

Logistic regression structure of the WCT Formula algorithm. Features (X*x*) are assigned a beta coefficient (β*x*) according to their effect on the binary outcome (i.e., VT or SWCT). The “constant” term (β_0_) represents the y-intercept for the least-squares regression line. The weighted sum predictor (X_β_) or VT probability (P) is calculated after integrating feature (X*x*) values derived from paired WCT and baseline ECG data. *ECG, electrocardiogram; PAC, percent amplitude change; SWCT, supraventricular tachycardia; VT, ventricular tachycardia; WCT, wide complex tachycardia.*

Logistic regression structure of the VT Prediction Model. Features (X*x*) are assigned a beta coefficient (β*x*) according to their effect on the binary outcome (i.e., VT or SWCT). The “constant” term (β_0_) represents the y-intercept for the least-squares regression line. The weighted sum predictor (X_β_) or VT probability (P) is calculated after integrating feature (X*x*) values derived from paired WCT and baseline ECG data. *ECG, electrocardiogram; SWCT, supraventricular tachycardia; VT, ventricular tachycardia; WCT, wide complex tachycardia.*

Logistic regression structure of the WCT Formula II algorithm. Features (X*x*) are assigned a beta coefficient (β*x*) according to their effect on the binary outcome (i.e., VT or SWCT). The “constant” term (β_0_) represents the y-intercept for the least-squares regression line. The weighted sum predictor (X_β_) or VT probability (P) is calculated after integrating feature (X*x*) values derived from paired WCT and baseline ECG data. *ECG, electrocardiogram; PTVAC, percent time voltage area change; SWCT, supraventricular tachycardia; VT, ventricular tachycardia; WCT, wide complex tachycardia*

Logistic regression structure of the Solo Model. Features (X*x*) are assigned a beta coefficient (β*x*) according to their effect on the binary outcome (i.e., VT or SWCT). The “constant” term (β_0_) represents the y-intercept for the least-squares regression line. The weighted sum predictor (X_β_) or VT probability (P) is calculated after integrating feature (X*x*) values derived from paired WCT and baseline ECG data. *ECG, electrocardiogram; PmonoTVA, percent monophasic time voltage area; SWCT, supraventricular tachycardia; VT, ventricular tachycardia; WCT, wide complex tachycardia.*

**Fig. 6:**
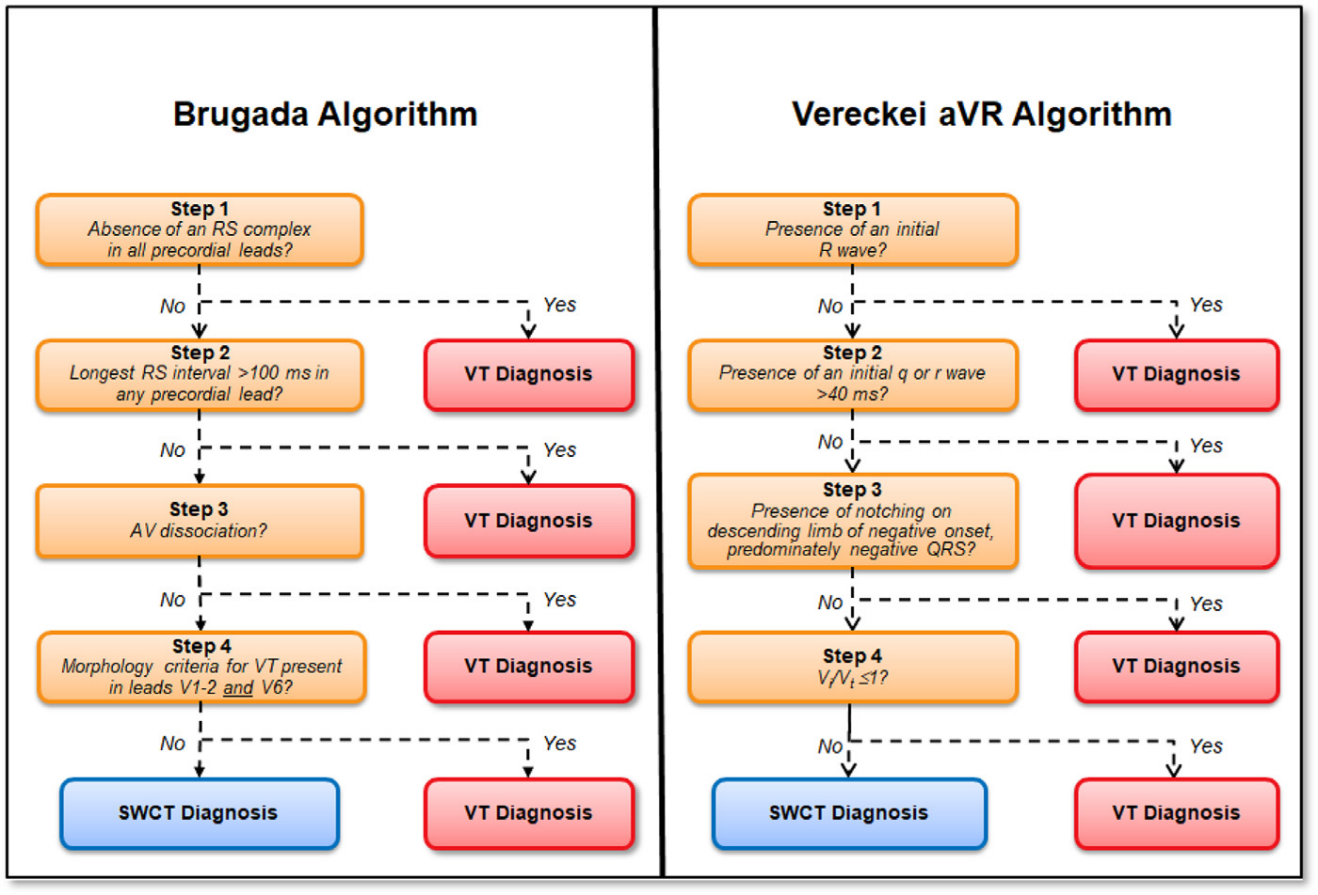
Brugada and Vereckei aVR algorithms.

Logistic regression structure of the Paired Model. Features (X*x*) are assigned a beta coefficient (β*x*) according to their effect on the binary outcome (i.e., VT or SWCT). The “constant” term (β_0_) represents the y-intercept for the least-squares regression line. The weighted sum predictor (X_β_) or VT probability (P) is calculated after integrating feature (X*x*) values derived from paired WCT and baseline ECG data. *ECG, electrocardiogram; SWCT, supraventricular tachycardia; VT, ventricular tachycardia; WCT, wide complex tachycardia*.
7. Fig. 6 illustrates the steps of the Brugada and Vereceki aVR algorithms, respectively (4,5).

**Fig. 7:**
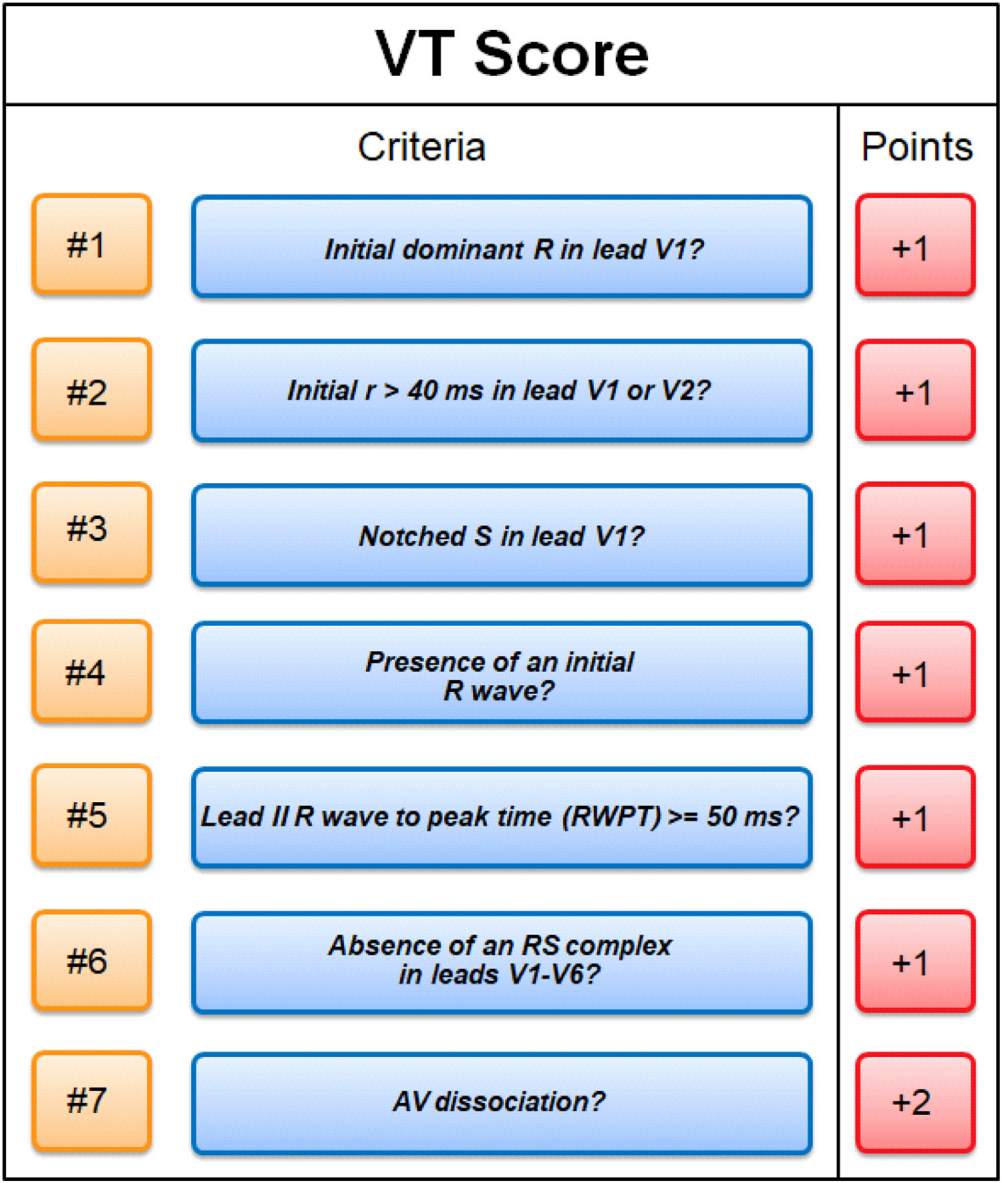
VT Score.

Illustration of the individual algorithm steps the electrophysiologists followed when manually applying the Brugada and Vereckei aVR algorithms. *SWCT, supraventricular tachycardia; VT, ventricular tachycardia*.
8. Fig. 7 illustrates the criteria (seven total) of the VT score (6). Each criterion was assigned a number (#1-7) to enable clear identification for data analysis.

**Fig. 8:**
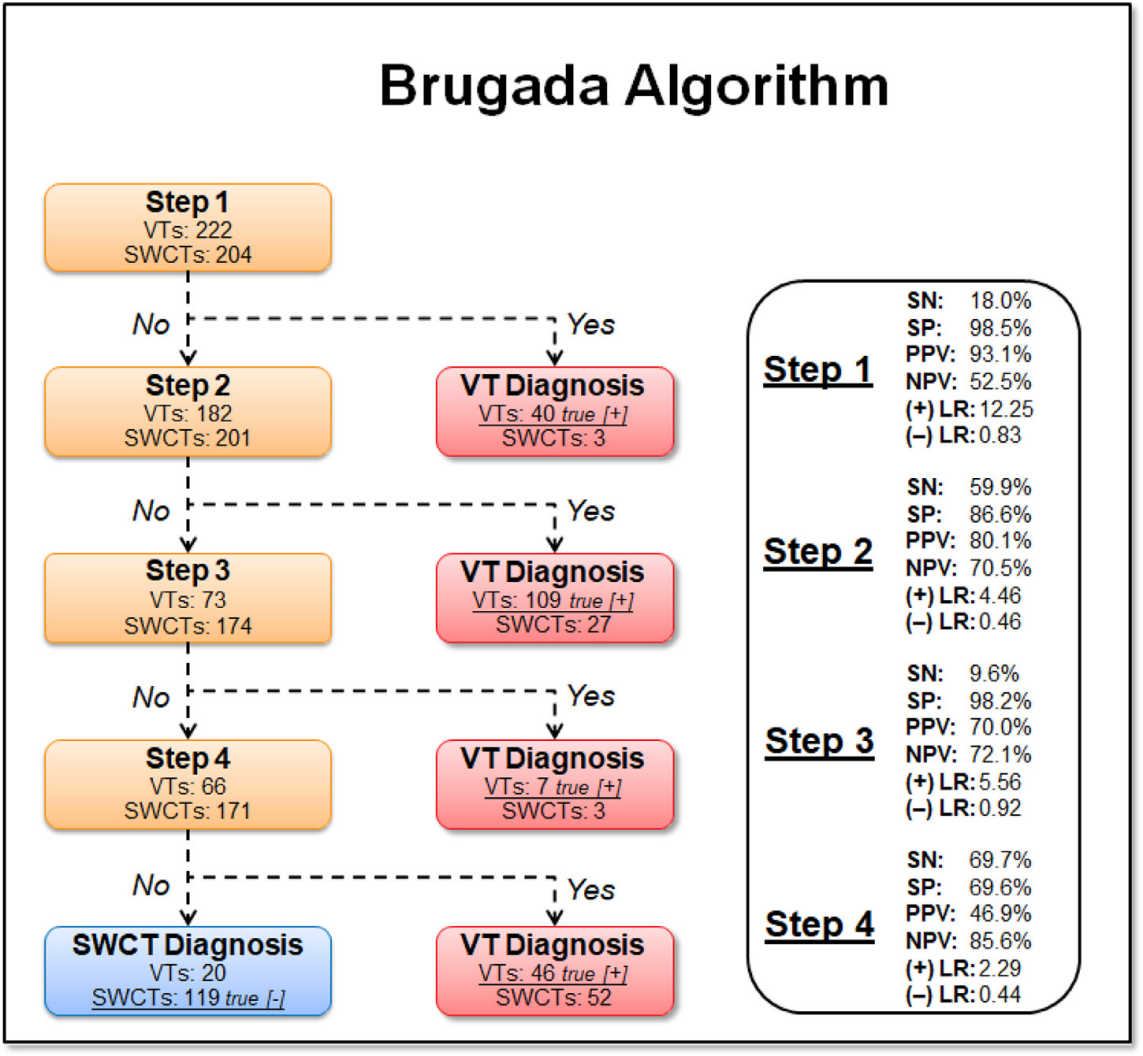
Diagnostic performance of Brugada algorithm.

Summary of VT Score criteria (seven total). Each criterion was assigned a number (#1 through 7) for data analysis purposes. The assigned point accrual if a criterion is met is also specified (+1 or +2 points). *AV, atrioventricular; RWPT, R wave to peak time.*
9. Fig. 8 details the diagnostic performance metrics for each step of the Brugada algorithm. Individual step performance was not comparable to that achieved in the original work (4).

**Fig. 9:**
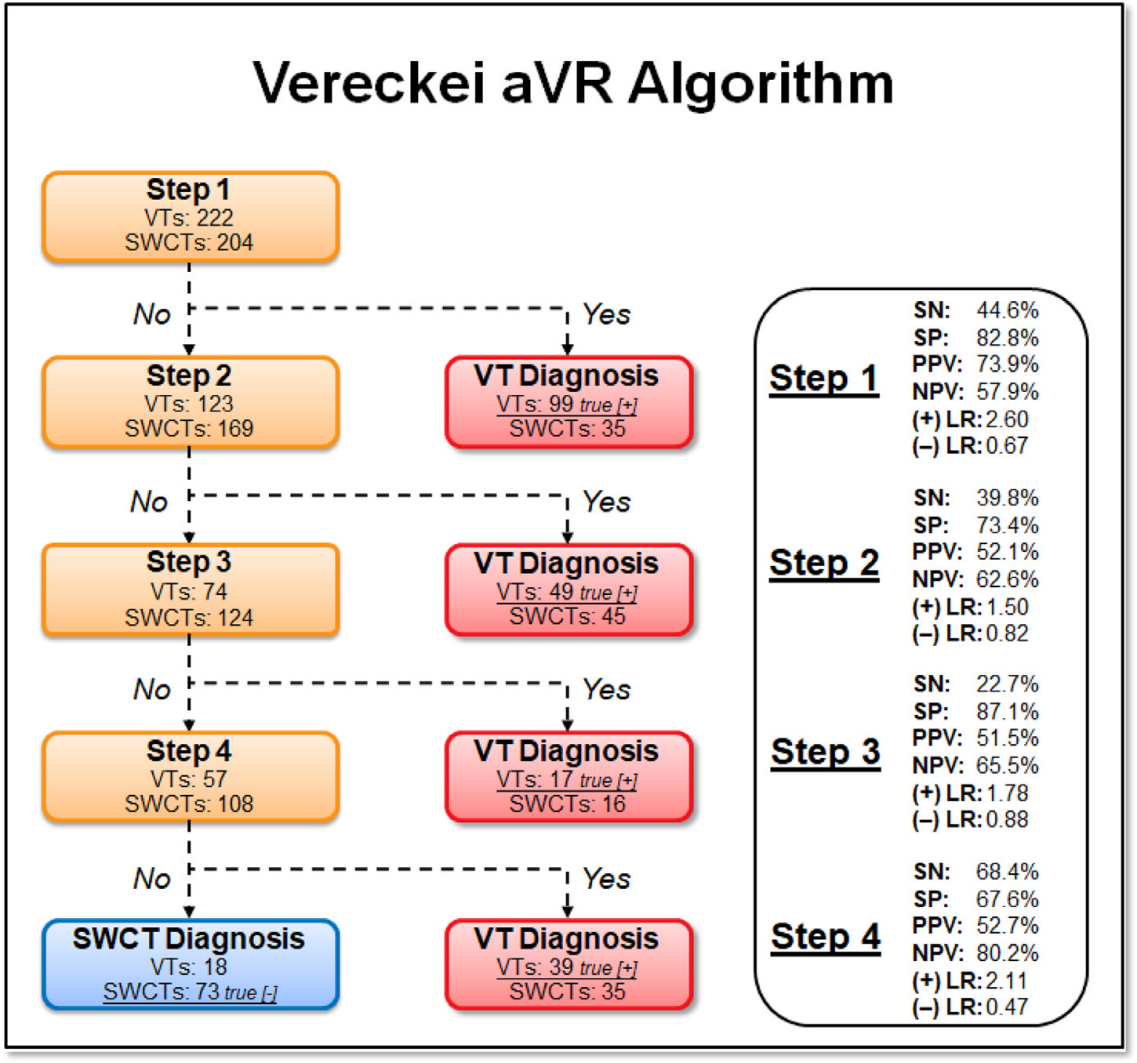
Diagnostic performance of Vereckei aVR algorithm.

Summary of the individual step performance for the Brugada algorithm, as applied by two electrophysiologists. *SN= sensitivity; SP= specificity; PPV= positive predictive value; NPV= negative predictive value; LR= likelihood ratio; (+) = positive; (-) = negative.*
10. Fig. 9 details the diagnostic performance metrics for each step of the the Vereckei aVR algorithm. Individual step performance was not comparable to that achieved in the original work (5).

Summary of the individual step performance for the Vereckei aVR algorithm, as applied by two electrophysiologists. *SN= sensitivity; SP= specificity; PPV= positive predictive value; NPV= negative predictive value; LR= likelihood ratio; (+)=positive; (-)=negative.*

## 4 Experimental Design, Materials and Methods

Recently, several studies have derived and evaluated the efficacy of automated algorithms for differentiating VT and SWCT [1,2,[7], [8], [9], [10]] Prior to this, decades of research focused on developing manual ECG interpretation algorithms for WCT differentiation [11,12].

In a two-part investigation [3], the diagnostic performance of three manual interpretation models (Brugada, Vereckei aVR, and VT Score) were directly compared to the performance of five automated interpretation models (WCT Formula I, WCT Formula II, VT Prediction Model, Solo Model, and Paired Model). In Part 1, two electrophysiologists, blinded to the clinical diagnosis, independently applied the Brugada algorithm, Vereckei aVR algorithm, and VT Score to selected WCT ECGs. Simultaneously, computerized data from paired WCT and baseline ECGs were incorporated into the five automated models. In Part 2, a direct comparison of the diagnostic performance between the automated algorithms and manual interpretation methods was conducted. Diagnostic performance metrics included accuracy, sensitivity, specificity, PPVs, NPVs, positive likelihood ratios, negative likelihood ratios and F1 scores.

The testing cohort for comparing manual and automated algorithms consisted of 213 WCTs (111 VT and 102 SWCT) from 104 patients, recorded at Barnes-Jewish Hospital in St. Louis between January 1, 2012, and December 31, 2014. ECGs were recorded in real-world clinical settings and were accessed from centralized data archives of an ECG interpretation software system (MUSE [*GE Healthcare*; Milwaukee, WI]). ECGs were standard 12-lead recordings with a paper speed of 25 mm/s and voltage calibration of 10 mm/mV and were required to satisfy standard WCT criteria above (QRS duration ≥ 120 ms and ventricular rate ≥ 100 beats per minute) and possess an official ECG overread interpretation of (i) ‘ventricular tachycardia’, (ii) ‘supraventricular tachycardia’, or (iii) ‘wide complex tachycardia’. Each clinical diagnosis (VT or SWCT) required a corroborating electrophysiology or implantable intracardiac device interrogation. Data related to clinical diagnosis, ECG laboratory interpretation and patient characteristics were recorded from the electronic medical record.

Five automated WCT differentiation models were used for comparative analysis: WCT Formula, WCT Formula II, VT Prediction Model, Solo Model, and Paired Model. These models were derived from 12-lead ECGs from Mayo Clinic Rochester and Mayo Clinic Health System of Southeastern Minnesota. Further details summarizing the patient cohorts used to derive and validate automated models has been previously reported [13,14]. Computerized ECG data from paired WCT and baseline ECGs were used to incorporate direct measurements or derived parameters into logistic regression models to classify VT or SWCT, or estimate the probability of VT (0.00%–99.99%).

The Brugada algorithm [4] uses four stepwise ECG criteria to differentiate between ventricular tachycardia (VT) and supraventricular tachycardia (SWCT). The steps include: (i) absence of an RS complex in all precordial leads, (ii) longest RS interval >100 ms in any precordial lead, (iii) presence of atrioventricular dissociation, and (iv) QRS morphology criteria for VT in leads V1–2 and V6. An affirmative answer to any step indicates VT, while negative responses across all steps suggest SWCT.

The Vereckei aVR algorithm [5] differentiates WCT using lead aVR. It includes: (i) presence of an initial R wave, (ii) initial r or q wave width >40 ms, (iii) notching on the descending limb of a negative QRS complex, and (iv) Vi/Vt ratio ≤1. As with the Brugada algorithm, a positive response to any step indicates VT.

The VT score [6] is a points-based algorithm evaluating seven VT-specific ECG criteria. Each criterion contributes 1 point, except for atrioventricular dissociation, which adds 2 points. A score of ≥1 indicates VT, while 0 points suggest SWCT. Since optimal diagnostic performance was reported at 1 point [6,15], this threshold was used for comparative analysis.

## Limitations

First, since this research was not conducted in actual clinical settings, the results may not fully reflect the diagnostic capabilities of manual and automated models in practice. Thus, comparisons among manual and automated algorithms might differ in real-world scenarios. Next, the three manual methods were assessed by only two electrophysiologists, so the data may not represent other manual techniques or professional groups, such as emergency medicine physicians or internal medicine residents. Lastly, the automated algorithms used a 50% VT probability threshold for diagnosis; results may vary with different thresholds. Similarly, the performance of the VT score, relative to the Brugada and Vereckei aVR algorithms would change with different point accrual thresholds.

## Ethics Statement

This research qualified for exemption status via the Institutional Review Board of Washington University in St. Louis, meeting such requirements for exemption under the guidelines set by the US Health and Human Services.

The authors have read and follow the ethical requirements for publication in Data in Brief and confirming that the current work does not otherwise involve animal experiments or any data collected from social media platforms.

## CRediT Author Statement

Sarah LoCoco, MD- conceptualization, formal analysis, investigation, resources, data curation, writing-original draft; Anthony H. Kashou, MD- conceptualization, methodology, software, writing- review and editing; Abhishek J. Deshmukh, MBBS**-** writing- review and editing, conceptualization; Samuel J. Asirvatham, MD- writing- review and editing, conceptualization; Christopher V. DeSimone MD, PhD- writing- review and editing, conceptualization; Krasimira M. Mikhova, MD- writing- review and editing; Sandeep S. Sodhi, MD, MBA- writing- review and editing; Phillip S. Cuculich, MD- writing- review and editing; Rugheed Ghadban, MD^-^ writing- review and editing, formal analysis, investigation, resources, data curation; Daniel H. Cooper, MD- writing- review and editing, formal analysis, investigation, resources, data curation; Thomas M. Maddox, MD, MSc- writing- review and editing, methodology; Peter A. Noseworthy, MD- writing- review and editing; Adam M. May, MD- conceptualization, supervision, project administration, funding acquisition, writing- original draft, software, validation, investigation.

## Data Availability

DataverseReplication Data for: WCT EP Answer Dataset (Original data).
